# Stunting Increases Influenza Virus Shedding Duration in Preschool/School-Aged Children

**DOI:** 10.1093/infdis/jiaf641

**Published:** 2025-12-18

**Authors:** Mia Q Zhu, Guillermina Kuan, Hannah E Maier, Roger Lopez, Abigail Shotwell, Miguel Plazaola, Sergio Ojeda, Nery Sanchez, Angel Balmaseda, Aubree Gordon

**Affiliations:** Department of Epidemiology, School of Public Health, University of Michigan, Ann Arbor, Michigan, USA; Sustainable Sciences Institute, Managua, Nicaragua; Centro de Salud Sócrates Flores Vivas, Ministerio de Salud, Managua, Nicaragua; Department of Epidemiology, School of Public Health, University of Michigan, Ann Arbor, Michigan, USA; Sustainable Sciences Institute, Managua, Nicaragua; Centro Nacional de Diagnóstico y Referencia, Ministerio de Salud, Managua, Nicaragua; Department of Epidemiology, School of Public Health, University of Michigan, Ann Arbor, Michigan, USA; Sustainable Sciences Institute, Managua, Nicaragua; Sustainable Sciences Institute, Managua, Nicaragua; Centro de Salud Sócrates Flores Vivas, Ministerio de Salud, Managua, Nicaragua; Sustainable Sciences Institute, Managua, Nicaragua; Centro de Salud Sócrates Flores Vivas, Ministerio de Salud, Managua, Nicaragua; Sustainable Sciences Institute, Managua, Nicaragua; Centro Nacional de Diagnóstico y Referencia, Ministerio de Salud, Managua, Nicaragua; Department of Epidemiology, School of Public Health, University of Michigan, Ann Arbor, Michigan, USA

## Abstract

The role of undernutrition on influenza virus shedding is unclear. We assessed stunting, an indicator of chronic undernutrition, as a predictor of shedding duration in children in the Household Influenza Cohort Study, in Managua, Nicaragua. Stunted children, aged 3–9 years, shed influenza longer than their nonstunted peers. In sub-group analysis, associations were driven by H3N2 and influenza B (IB) infections. Analysis of cycle threshold (Ct) values showed slower clearance of IB viruses in stunted children. These findings suggest undernutrition may prolong influenza shedding, with implications for transmission dynamics and control strategies in low- and middle-income countries where stunting remains prevalent.

## Teaser

Preschool/school-aged children with stunting shed influenza virus longer than their nonstunted counterparts. This association was strongest for influenza B viruses and may be driven by slower clearance of these viruses in children with stunting.

Influenza is common in childhood and adolescence and is an important contributor to school absenteeism and childhood morbidity and mortality globally [1]. Nevertheless, influenza burden disproportionately affects low-to-middle-income countries (LMICs) where most influenza-related deaths occur [1]. This excess burden can, in part, be attributed to pre-existing health conditions prevalent in these countries including malnutrition [2]. Stunting, or childhood growth faltering, is an accessible and commonly utilized marker of chronic undernutrition [3]. Despite policies to reduce undernutrition globally, stunting still affects between 6% and >50% of children in LMICs [4].

Undernutrition can inhibit immune function through various mechanisms, including an impaired gut-barrier and a delayed or reduced immune response [2]. While there is some epidemiological evidence that undernutrition is related to greater incidence and severity of influenza infection [2, 5], the role undernutrition may have in influenza transmission remains unclear. Viral shedding duration is commonly used to define period of infectivity and is vital to understanding influenza transmission [6]. Assessing factors that may impact viral shedding is especially important in children, who are important drivers of community and household influenza transmission [1]. We, therefore, utilized a household influenza cohort to examine stunting as a predictor of influenza shedding duration in children.

## METHODS

### Study Description

The Household Influenza Cohort Study (HICS) is an ongoing household-based cohort study in Managua, Nicaragua established in June 2017 [7]; end of follow-up for this study was May 2025. Participants reported to the study clinic when they developed respiratory symptoms and were tested for influenza by RT-PCR. Upon a positive test (Cycle threshold [Ct] value <40), an intensive monitoring period (IMP) of approximately one month was initiated, with respiratory samples collected from all household members regardless of symptoms every few days up to five samples. We utilized household samples collected from children and adolescents aged <19 years. The study was approved by the institutional review boards of the Nicaraguan Ministry of Health and the University of Michigan. Written informed consent was obtained from a parent/guardian, with verbal assent from children aged ≥6 years.

### Shedding Duration

Viral shedding duration was defined from successive RT-PCR results and analyzed using survival models accounting for right- and interval-censoring, as described previously [7, 8]. For index cases, earliest shedding start date was set two days before symptom onset; for secondary cases, the first positive test date was used. Shedding end was defined as the first negative sample after the last positive (or right-censored if not observed).

### Stunting

Height was measured at enrollment, annual surveys, and clinic visits. To assess stunting status directly prior or during infection, only measurements from the year prior to an infection episode were considered and the median was used if multiple were available. Height-for-age *z*-scores were calculated using World Health Organization (WHO) standards [9], and stunting was defined as *z* < −2.

### Covariates

Age at infection was categorized as 0–2 (infants/toddlers), 3–9 (preschool/school-aged children), and 10–18 years (adolescents), corresponding to critical periods for stunting development [3]. Body mass index (BMI) for age and sex *z*-score was calculated per WHO standards. Symptoms were recorded in daily journals during IMPs and at clinic visits. Asymptomatic/mild infection was defined as <2 respiratory symptoms not including fever and moderate/severe illness as lower respiratory symptoms or hospitalization. Recent vaccination was defined as receipt ≥14 days and ≤180 days prior to infection.

### Statistical Analysis

Parametric accelerated failure time models (AFT) with lognormal distribution and robust standard errors, accounting for household clustering and shedding duration censoring, were used to estimate shedding duration and event time ratios (ETR) with 95% confidence intervals (CI). To assess effect measure modification, models were stratified by age and infection type. To account for confounding and extraneous variation of shedding duration, we further adjusted for sex, BMI, vaccination status, and season. Sensitivity analysis was performed by restricting to secondary infections. Risk ratios for moderate/severe illness were estimated using generalized estimating equations with a Poisson distribution and a log link. To assess viral load over time by stunting status, Ct values from RT-PCR tests, considered as semiquantitative measures of viral load, were modeled using generalized additive models with a cubic spline for time since first symptom and first positive sample for index and secondary cases, respectively. Analyzes were conducted in SAS 9.4 and R 4.5.1 (survival, geepack).

## RESULTS

Between June 2017 and May 2025, 529 cohort households initiated 1029 intensive monitoring periods. Across these monitoring periods, 2186 participants (54% children) contributed 22 419 swabs (a median of 5 swabs per person per period) resulting in 1423 PCR-confirmed influenza infections in children. After excluding cases with coinfections and missing height, 1353 infections (779 index and 574 secondary cases) from 851 children comprised our analytic sample. These infections, collected over 6 influenza seasons, consisted of 885 (65%) influenza A (34% H1N1pdm and 65% H3N2) and 468 (35%) influenza B (28% Yamagata and 70% Victoria) infections (Supplementary Figure 1). The median age of children with influenza was 8.0 years (IQR: 4.5–12.0) and 49% were female. Overall, 6% (*n* = 87) of children met criteria for stunting. Children with stunting were similar to their nonstunted peers with respect to sex, age and other covariates (Supplementary Table 1).

### Overall Viral Shedding

We first examined overall patterns of viral shedding across age, infection type, and clinical presentation. The mean predicted shedding duration across all infections was 8.3 days (SD: 5.6). Shedding duration was longer in younger children and when infection was symptomatic but did not differ by child's sex and recent vaccination status (Supplementary Figure 2). Mean predicted shedding duration for infants/toddlers, preschool/school-aged children, and adolescents were, respectively, 8.3, 7.4, and 5.9 days (Supplementary Figure 2A). After adjusting for age, symptomatic infections were associated with 74% (95% CI: 47%, 106%) longer shedding duration compared with asymptomatic/mild infections. Shedding duration did not differ substantively by influenza type/influenza A subtype or severity (Supplementary Figure 3).

### Viral Shedding by Stunting Status

**Figure 1.**
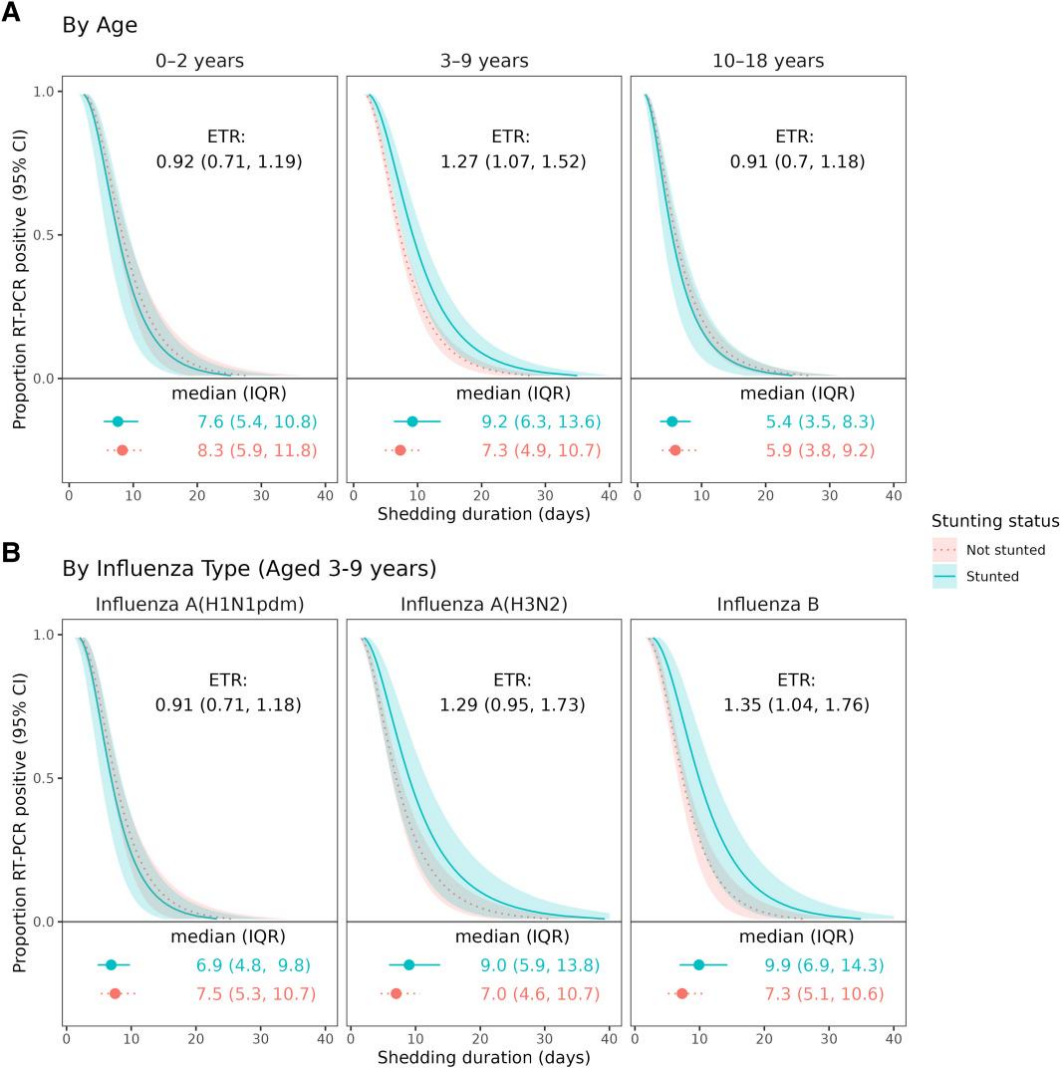
Influenza shedding duration by stunting status from crude models. (*A*), stratified by age and (*B*) virus type/subtype in children aged 3–9 y during infection. Event time ratios and predicted shedding durations are from accelerated failure time models with a lognormal distribution and robust standard errors. ETR >1 indicates longer predicted viral shedding for stunted compared with nonstunted children. Shaded regions represent 95% confidence intervals. Estimated median and interquartile range shedding durations are displayed graphically and in text below each figure.

Preschool/school-aged children with stunting shed influenza virus for a significantly longer duration compared with those without stunting. The median shedding duration was 9.2 (IQR 6.3, 13.6) days for stunted preschool/school-aged children and 7.3 (IQR 4.9, 10.7) days for nonstunted children (Table 1, Figure 1*A*). In adjusted models, stunted preschool/school-aged children experienced 26% (95% CI: 5%, 51%) longer shedding duration (Table 1) than nonstunted peers. This association was stronger for infections by H3N2 and influenza B viruses (Figure 1*B*).

**Table 1.** Accelerated Failure Time Models of Stunting and Shedding Duration in Children and Adolescents, by Influenza Virus Type in the Household Influenza Cohort Study 2017–2024

	Influenza Infections	Predicted Shedding Duration, Median (IQR)^a^	Unadjusted ETR (95% CI)^a^	Adjusted^b^ ETR (95% CI)
All Viruses	…	…	…	…
Stunting	…	…	…	…
No	1266	6.9 (4.6, 10.4)	Reference	Reference
Yes	87	7.2 (4.8, 10.8)	1.04 (.87, 1.24)	1.06 (.91, 1.24)
By Influenza Type	…	…	…	…
A Viruses	…	…	…	…
Stunting	…	…	…	…
No	829	6.9 (4.5, 10.4)	Reference	Reference
Yes	56	6.8 (4.5, 10.4)	.99 (.81, 1.22)	1.01 (.85, 1.21)
B Viruses	…	…	…	…
Stunting	…	…	…	…
No	437	7.0 (4.8, 10.3)	Reference	Reference
Yes	31	7.9 (5.4, 11.7)	1.13 (.89, 1.43)	1.15 (.9, 1.46)
Stunting by Age, y	…	…	…	…
0–2	…	…	…	…
No	187	8.3 (5.9, 11.8)	Reference	Reference
Yes	17	7.6 (5.4, 10.8)	.92 (.71, 1.19)	.96 (.69, 1.34)
3–9	…	…	…	…
No	606	7.3 (4.9, 10.7)	Reference	Reference
Yes	32	9.2 (6.3, 13.6)	1.27 (1.07, 1.52)	1.26 (1.05, 1.51)
10–18	…	…	…	…
No	473	5.9 (3.8, 9.2)	Reference	Reference
Yes	38	5.4 (3.5, 8.3)	.91 (.70, 1.18)	.93 (.71, 1.20)

Abbreviations: AFT, accelerated failure time; CI, confidence interval; ETR, event time ratio; IQR, interquartile range.

^a^From an accelerated failure time model with a lognormal distribution and robust standard errors. ETR >1 indicates longer predicted viral shedding for stunted compared with nonstunted children.

^b^Adjusted for influenza type, age at infection, sex, weight category, recent vaccination, influenza season; in models stratified by infection type, influenza type was removed as an adjustment variable.

In analysis of secondary cases, associations of stunting and influenza shedding duration were stronger. Stunted preschool/school-aged children experienced an adjusted 71% (95% CI: 20%, 142%) longer influenza shedding compared with nonstunted children (Supplementary Table 2). Similarly, associations were limited to H3N2 and influenza B infections, with stunted preschool/school-aged children experiencing 48% (95% CI: 2%, 115%) and 90% (95% CI: 17%, 209%) longer shedding, respectively, compared with nonstunted children (Supplementary Figure 4).

Stunting status was not related to infection severity. Mean minimum Ct values for stunted versus nonstunted children were similar (26 and 25.3, respectively), and stunted children and adolescents were not substantively more likely to experience moderate/severe clinical outcomes during their illness (Supplementary Table 3). Nevertheless, the rate of Ct increase after influenza B infection was slower for children with stunting (Supplementary Figure 5).

## DISCUSSION

We found that stunted school-aged children shed influenza A H3N2 and influenza B viruses longer than their nonstunted counterparts. To our knowledge, this is the first study to assess stunting as a predictor of influenza shedding duration.

While studies on undernutrition and viral shedding are sparse, existing research on malnutrition, more generally, and viral shedding support our findings. A recent study found that poor nutritional status in older adults was associated with longer shedding of SARS-CoV-2 virus [10]. Furthermore, we have previously found that adults with obesity shed influenza A virus longer than adults without obesity [7]. Although obesity occurs from over-nutrition rather than undernutrition, both forms of malnutrition are associated with immune deficiencies [11]. While both obesity and undernutrition were linked to prolonged shedding, their respective associations were observed only in adults and school-aged children, suggesting that malnutrition through under- and over-nutrition may influence immune function differently depending on immunological maturity. Adolescents and adults, who have a more robust immune response compared with children, may be more resilient to the adverse effects of undernutrition. Alternatively, indicators of malnutrition may differ by age: stunting, which is irreversible beyond childhood, may best capture chronic malnutrition in children, while measures of adiposity like BMI may better reflect malnutrition in adults. Future studies should consider assessing wasting, or very low weight-for-height, as a predictor of influenza shedding as it better reflects acute malnutrition to further clarify the short- and long-term effects of undernutrition on immune function.

Stunting was positively associated with prolonged shedding, but not with illness severity or viral load, suggesting undernutrition may affect mechanisms related to viral clearance rather than viral replication. This finding is consistent with a challenge study that found slower clearance of influenza A titers 9 days postinfection in mice fed with a very low protein diet compared with mice fed an adequate protein diet, despite similar viral titer levels soon after infection [12]. The exact mechanisms by which malnutrition may impair viral clearance are unclear. Malnutrition causes deficiencies in zinc, vitamin A, proteins, and dietary fats, which are essential for T- and B-cell responses and for maintaining the gut microbiota, thereby inhibiting immune function [2, 11]. Alternatively, stunted children are at increased risk of impaired lung growth and function [13], which can reduce the mucociliary viral clearance and increase airway inflammation, resulting in prolonged viral shedding [14].

Stunting predicted longer shedding of influenza B viruses, marginally longer H3N2 shedding and was not related to H1N1pdm. The specificity of this association suggests undernutrition may influence viral shedding through viral and host response mechanisms specific to influenza B and, to a lesser degree, H3N2. One intriguing possibility involves differences in baseline antibody levels between virus subtypes/types. Assuming undernutrition reduces production of antibodies to all viruses, higher baseline H1N1 antibody levels in these children could preclude the need for a new adaptive humoral response to clear the virus. Therefore, the mechanism by which undernutrition inhibits viral clearance would not impact H1N1pdm shedding. To explain these mechanisms, future research on the role of undernutrition in regulating specific viral and host responses, including analysis of antigenic drift and cytokine, antibody, and cell-mediated responses, is warranted.

Stunting did not predict longer influenza shedding in adolescents. Since their period for linear growth has mostly passed, stunting may not be a good indicator of current nutritional status in older children. The lack of an association in adolescents suggests that only undernutrition at the time of infection may play a role in influenza shedding duration. Furthermore, no association between stunting and influenza shedding was observed in infants and toddlers, a period when stunting may be unstable due to catch-up growth [3]. This finding highlights a critical period for public health intervention. Reducing stunting in toddlers—before growth patterns become fixed—may be an important strategy for decreasing influenza transmission in LMICs, particularly since preschool/school-aged children are primary drivers of transmission in both community and household settings [1].

Our study has several strengths. First, height and viral shedding were objectively measured. Second, intensive household sampling of household contacts was conducted irrespective of symptoms, allowing for detection of asymptomatic infections. Third, the prospective design with repeated household follow-up enabled detailed characterization of shedding dynamics, an approach rarely available. Finally, the relative homogeneity of the study population reduces the likelihood of residual confounding.

This study has several limitations. First, the exact shedding duration was estimated for many children, and although our model accounts for censoring, misclassification remains possible. Second, shedding duration is not a perfect proxy of infectivity. Finally, residual confounding through common causes of malnutrition and shedding duration is possible. Nevertheless, limitations in causal interpretations do not reduce the utility of these findings for modeling influenza transmission dynamics, especially in communities where stunting is common.

In conclusion, we found that preschool/school-aged children with stunting shed influenza virus substantially longer than nonstunted peers. These findings highlight the importance of addressing chronic undernutrition in childhood, not only for individual health but also for its potential impact on community-level transmission. The intensive prospective household design strengthens confidence in these findings and highlights the value of such cohorts in understanding the interplay of chronic and infectious diseases.

## Supplementary Material

jiaf641_Supplementary_Data

## Supplementary Data

Supplementary materials are available at The Journal of Infectious Diseases online (http://jid.oxfordjournals.org/). Supplementary materials consist of data provided by the author that are published to benefit the reader. The posted materials are not copyedited. The contents of all supplementary data are the sole responsibility of the authors. Questions or messages regarding errors should be addressed to the author.
