# Supplementary material for: Stunting Increases Influenza Virus Shedding Duration in Preschool/School-Aged Children
Source: J Infect Dis. 2025 Dec 18;233(3):e777–81. doi: 10.1093/infdis/jiaf641 (PMC13016895; doi:10.1093/infdis/jiaf641)
Supplement: jiaf641_Supplementary_Data [file jiaf641_supplementary_data.docx]

Table S1. Characteristics of influenza infections in the Household Influenza Cohort Study.

|  | Secondary infections, N (%) | | |
| --- | --- | --- | --- |
|  | Total^1^ | Not Stunted | Stunted |
|  |  |  |  |
| All | 1353 (100%) | 1266 (94%) | 87 (6%) |
| Age, y |  |  |  |
| 0-2 | 204 (15%) | 187 (92%) | 17 (8%) |
| 3-9 | 638 (47%) | 606 (95%) | 32 (5%) |
| 10-18 | 511 (38%) | 473 (93%) | 38 (7%) |
| Sex |  |  |  |
| F | 666 (49%) | 620 (93%) | 46 (7%) |
| M | 687 (51%) | 646 (94%) | 41 (6%) |
| Influenza type |  |  |  |
| Influenza A | 885 (65%) | 829 (94%) | 56 (6%) |
| Influenza B | 468 (35%) | 437 (93%) | 31 (7%) |
| Influenza A subtype |  |  |  |
| H3N2 | 577 (43%) | 537 (93%) | 40 (7%) |
| H1N1pdm | 297 (22%) | 283 (95%) | 14 (5%) |
| Symptomatic |  |  |  |
| No | 111 (9%) | 105 (95%) | 6 (5%) |
| Yes | 1242 (91%) | 1161 (93%) | 81 (7%) |
| Moderate/severe illness |  |  |  |
| No | 1286 (95%) | 1205 (94%) | 81 (6%) |
| Yes | 67 (5%) | 61 (91%) | 6 (9%) |
| Recent vaccination^2^ |  |  |  |
| No | 1300 (96%) | 1217 (94%) | 83 (6%) |
| Yes | 53 (4%) | 49 (92%) | 4 (8%) |
|  |  |  |  |
| ^1^ Totals may not match due to missing in covariates. % for Total is out of total participants, 1,353; % for not stunted/stunted is out of row totals.  ^2^ Vaccination ≥ 14 days and ≤180 days before infection | | | |

Table S2. Accelerated Failure Time models of stunting and shedding duration **in secondary household infections of children and adolescents,** by influenza virus type in the Household Influenza Cohort Study 2017-2024.

|  | Influenza infections | Predicted shedding duration, median (IQR)^1^ | Unadjusted ETR (95% CI)^1^ | Adjusted^2^ ETR (95% CI) |
| --- | --- | --- | --- | --- |
|  |  |  |  |  |
| All Viruses |  |  |  |  |
| Stunting |  |  |  |  |
| No | 530 | 5.9 (3.7, 9.3) | Reference | Reference |
| Yes | 44 | 6.5 (4.1, 10.3) | 1.10 (0.85, 1.45) | 1.14 (0.90, 1.45) |
|  |  |  |  |  |
| By Influenza Type |  |  |  |  |
| A Viruses |  |  |  |  |
| Stunting |  |  |  |  |
| No | 341 | 5.9 (3.7, 9.5) | Reference | Reference |
| Yes | 29 | 6.1 (3.8, 9.8) | 1.03 (0.80, 1.33) | 1.07 (0.83, 1.37) |
| B Viruses |  |  |  |  |
| Stunting |  |  |  |  |
| No | 189 | 5.8 (3.7, 9.0) | Reference | Reference |
| Yes | 15 | 7.3 (4.7, 11.4) | 1.27 (0.79, 2.04) | 1.26 (0.82, 1.93) |
|  |  |  |  |  |
| By Age, y |  |  |  |  |
| 0-2 |  |  |  |  |
| Stunting |  |  |  |  |
| No | 74 | 7.5 (4.9, 11.4) | Reference | Reference |
| Yes | 7 | 6.8 (4.4, 10.4) | 0.91 (0.52, 1.60) | 0.90 (0.50, 1.6) |
| 3-9 |  |  |  |  |
| Stunting |  |  |  |  |
| No | 242 | 6.2 (4.0, 9.8) | Reference | Reference |
| Yes | 13 | 10.0 (6.4, 15.8) | 1.61 (1.19, 2.19) | 1.71 (1.20, 2.42) |
| 10-18 |  |  |  |  |
| Stunting |  |  |  |  |
| No | 214 | 5.0 (3.1, 7.9) | Reference | Reference |
| Yes | 24 | 5.0 (3.1, 7.9) | 1.00 (0.72, 1.40) | 1.00 (0.70, 1.43) |
|  |  |  |  |  |
| Abbreviations: AFT, accelerated failure time; CI, confidence interval; ETR, event time ratio; IQR, interquartile range  ^1^ From an accelerated failure time model with a lognormal distribution and robust standard errors  ^2^ Adjusted for influenza type, age at infection, sex, weight category, recent vaccination, influenza season | | | | |

Table S3. Risk of moderate/severe illness by stunting status.

|  |  | Moderate/Severe | |  | Risk Ratios of Moderate/Severe^1^ | |
| --- | --- | --- | --- | --- | --- | --- |
|  | N infections | N | % |  | Unadjusted | Adjusted^2^ |
|  |  |  |  |  |  |  |
| All |  |  |  |  |  |  |
| Not stunted | 1,266 | 61 | 4.8 |  | Reference | Reference |
| Stunted | 87 | 6 | 6.9 |  | 1.43 (0.57, 3.62) | 1.48 (0.59, 3.70) |
|  |  |  |  |  |  |  |
| ^1^ From generalized estimating equation models with a Poisson distribution and a log link. Outcome was defined as 0 for asymptomatic/mild infection and 1 for moderate/severe infection.  ^2^ Adjusted for age, sex, influenza type, and vaccination status. | | | | | | |


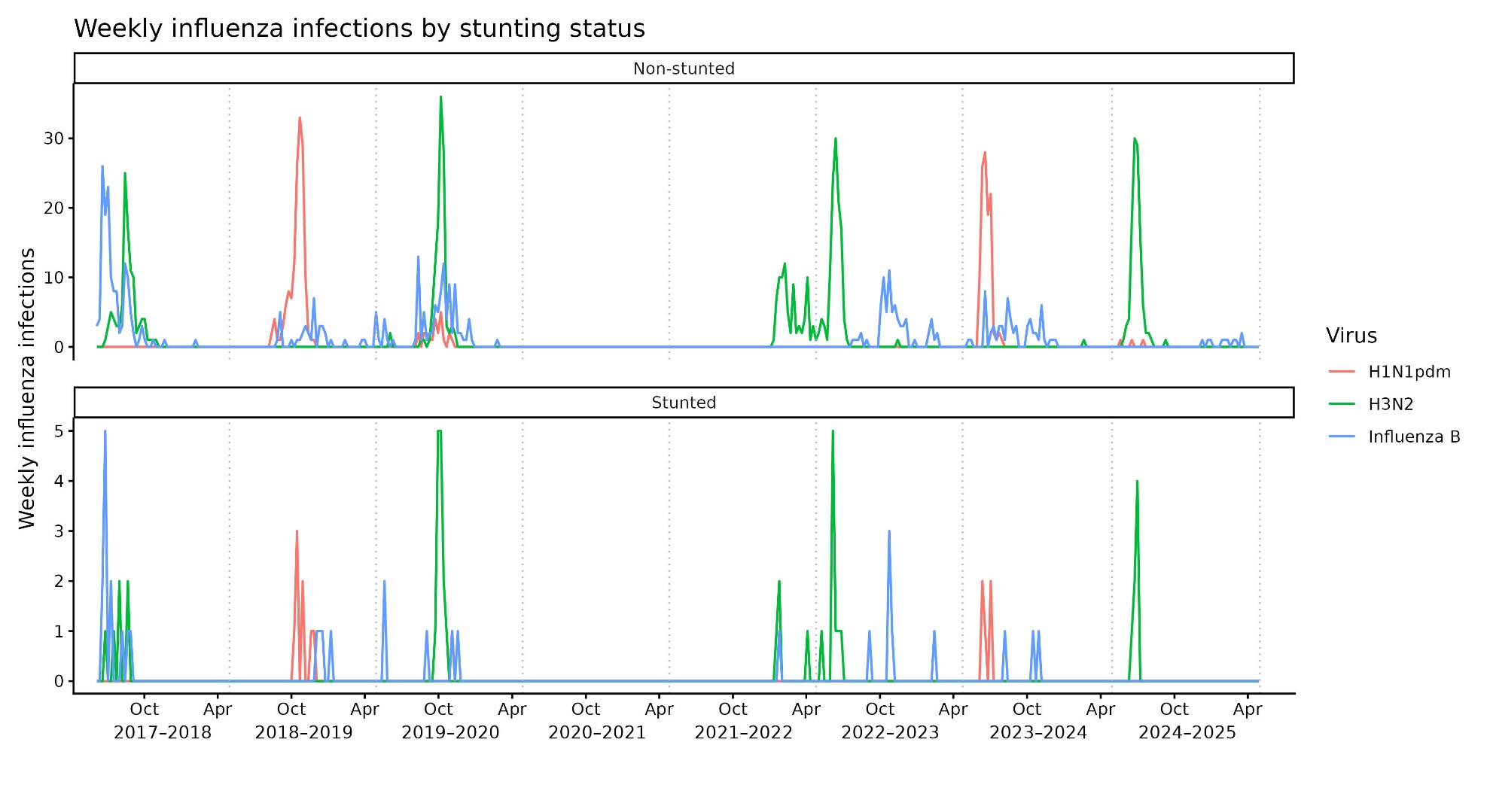


Figure S1. Weekly influenza infection counts by stunting status and virus.


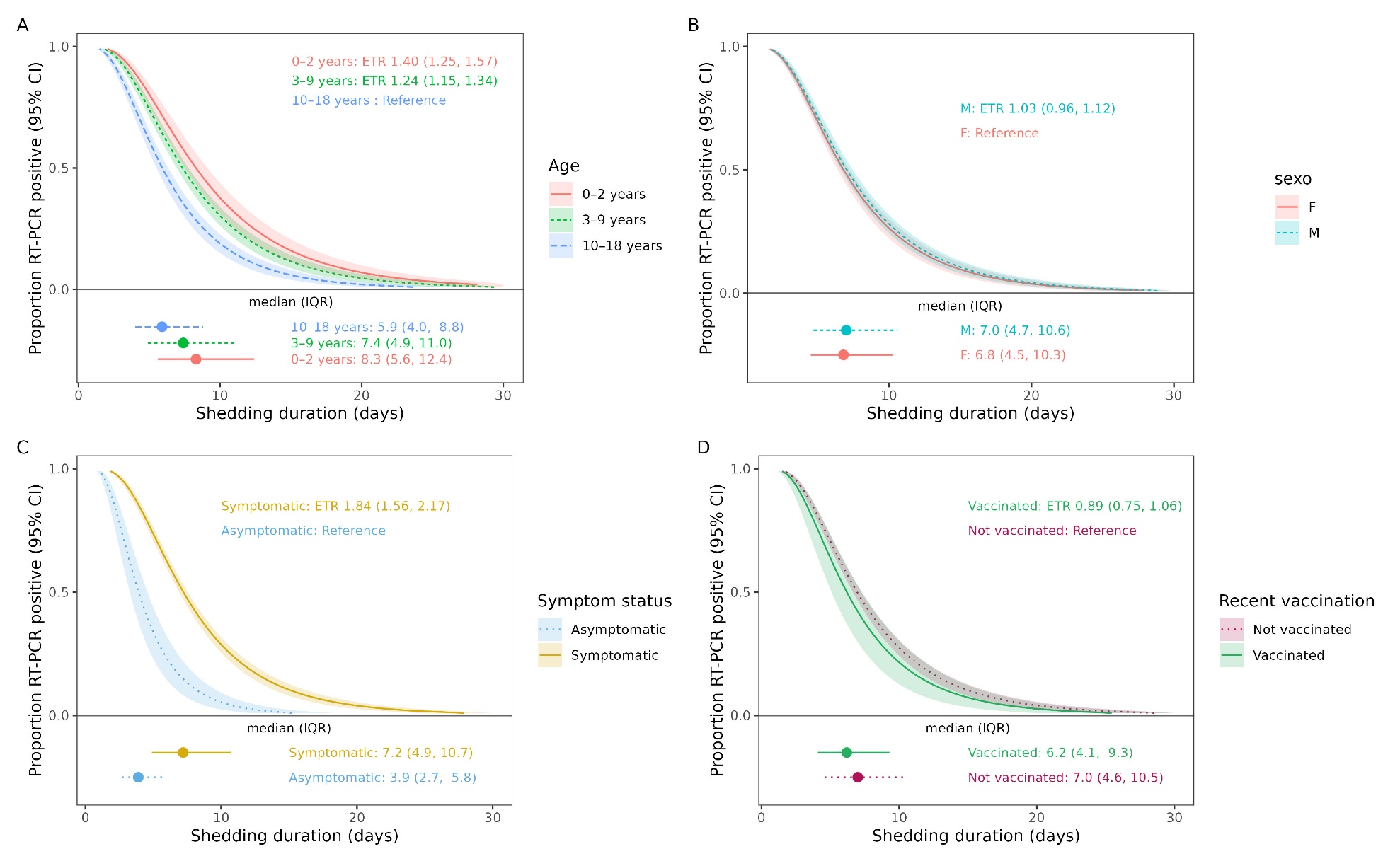


Figure S2. Predicted shedding duration from crude accelerated failure time models with a lognormal distribution and robust standard errors by A) age, B) sex, C) symptomatic infection, and D) recent vaccination.


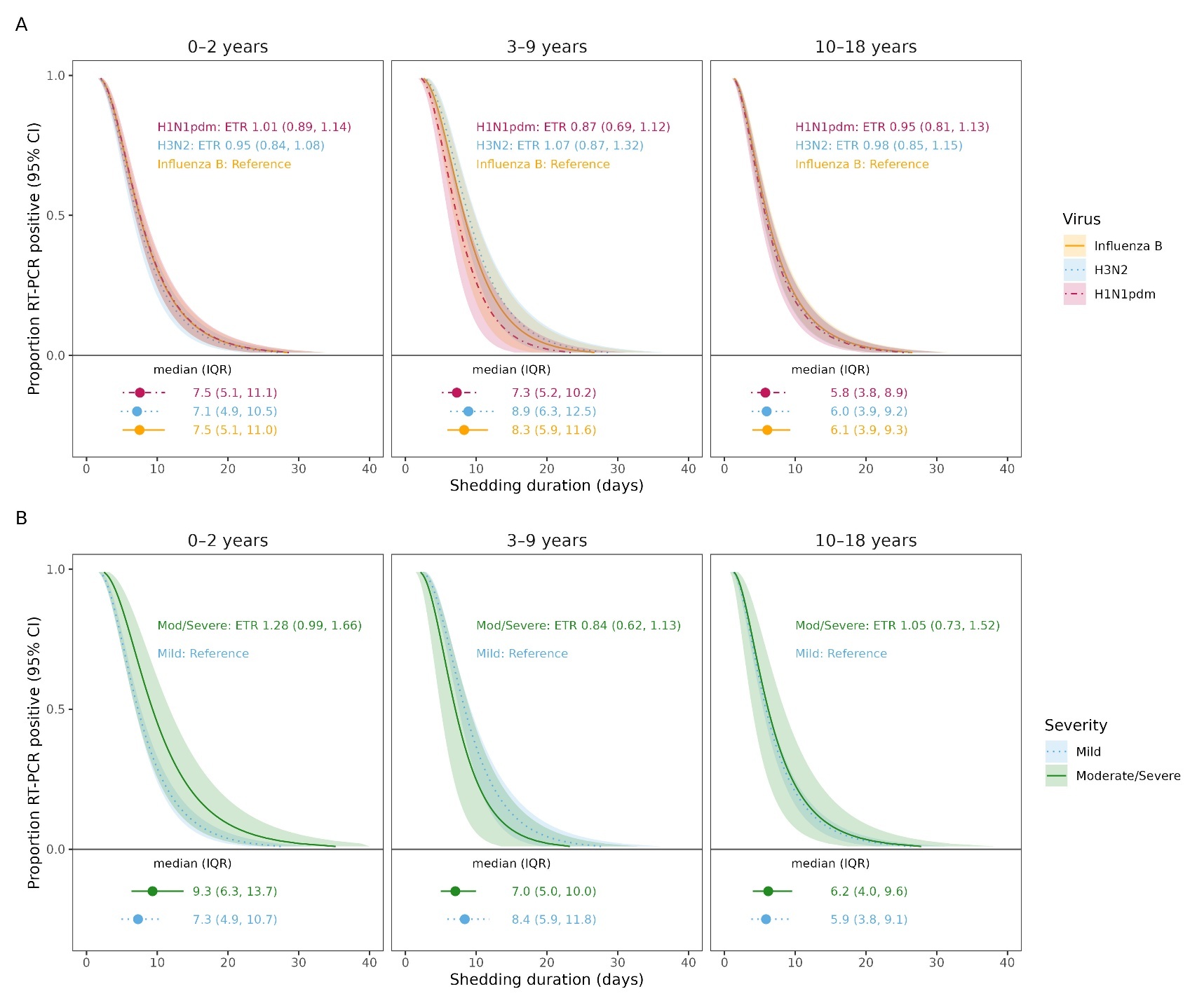


Figure S3. Predicted shedding duration from accelerated failure time models with a lognormal distribution and robust standard errors by infection type/subtype and severity, stratified by age.


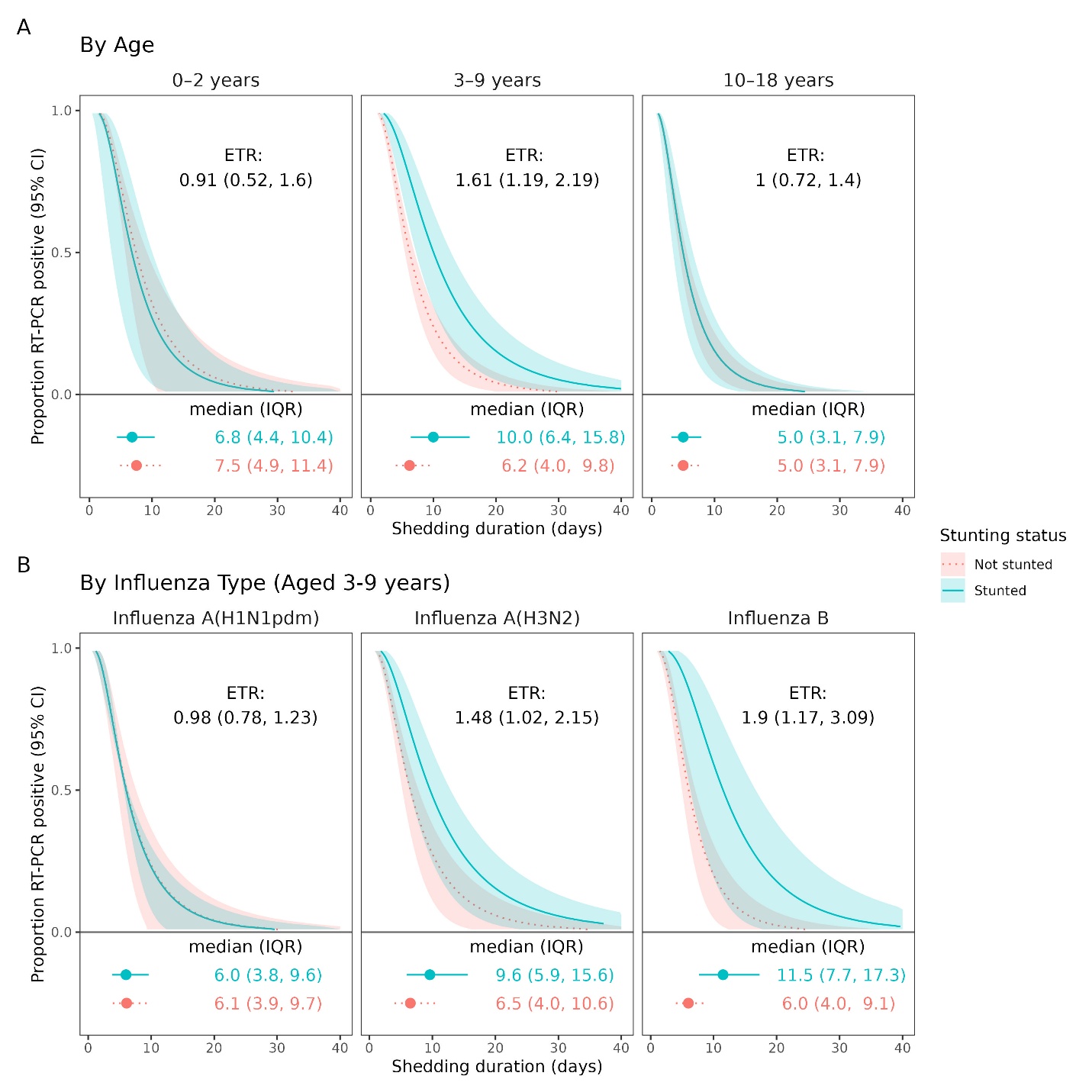


Figure S4. Influenza shedding duration by stunting status in secondary cases from crude models, A) stratified by age and B) virus type/subtype in children aged 3-9 years during infection. Event time ratios (ETR) and predicted shedding durations are from accelerated failure time models with a lognormal distribution and robust standard errors. Shaded regions represent 95% confidence intervals. Estimated median and interquartile range (IQR) shedding durations are displayed graphically and in text below each figure.


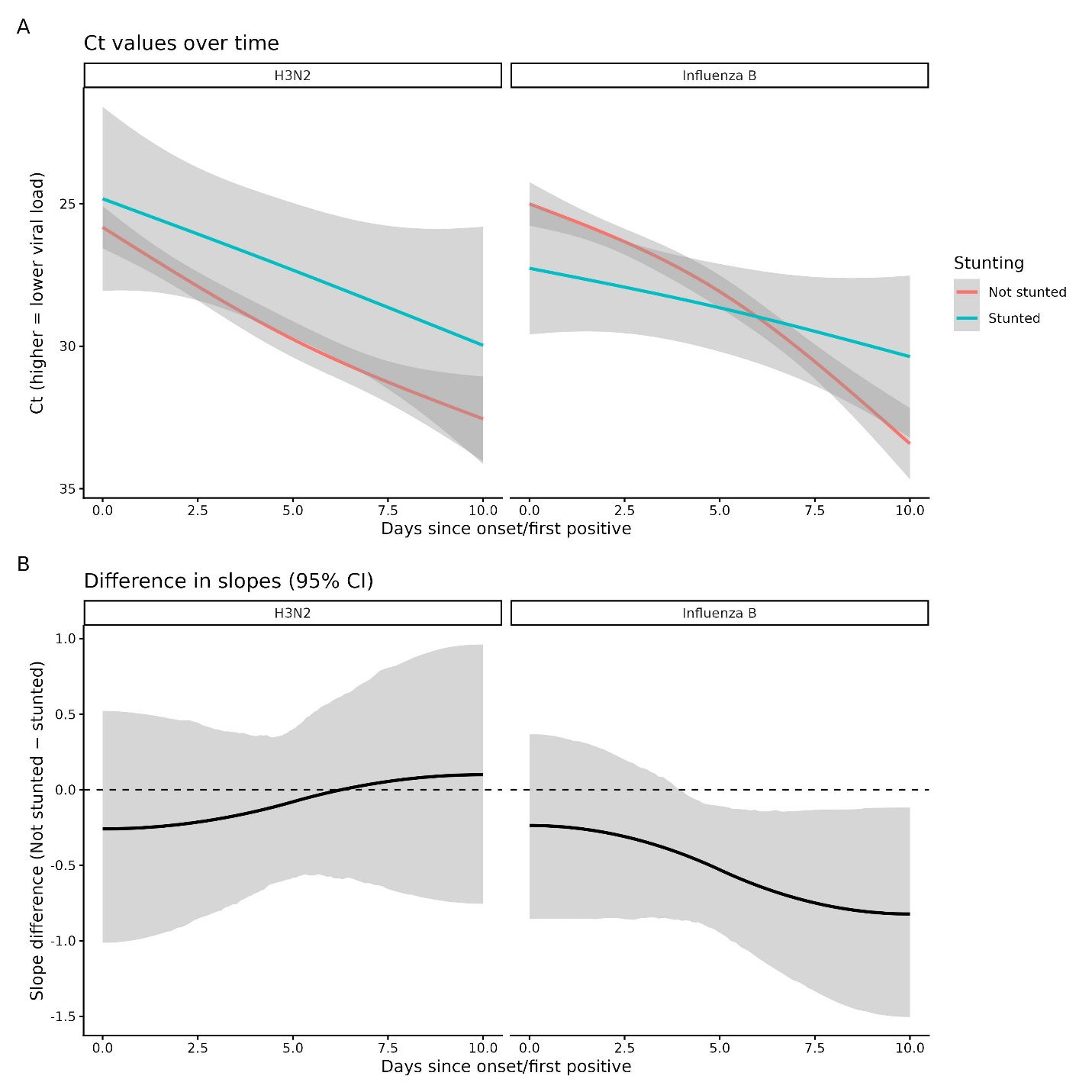


Figure S5. A) Ct values over time by stunting status. From gaussian generalized additive models with a cubic spline term (with basis dimension k =3). For index cases, days were defined as days from date of first symptom. For secondary cases, days were defined as days from date of first positive RT-PCR test. B) Instantaneous slope difference between stunted and non-stunted children. Negative values indicate rate of increase in Ct values (rate of decrease in viral load) was slower for stunted children compared to their non-stunted peers.
